# The CNP signal is able to silence a supra threshold neuronal model

**DOI:** 10.3389/fncom.2015.00044

**Published:** 2015-04-28

**Authors:** Francesca Camera, Alessandra Paffi, Alex W. Thomas, Francesca Apollonio, Guglielmo D'Inzeo, Frank S. Prato, Micaela Liberti

**Affiliations:** ^1^Department of Information Engineering, Electronics and Telecommunications, “Sapienza” University of RomeRome, Italy; ^2^Bioelectromagnetics Group, Imaging Program, Lawson Health Research InstituteLondon, ON, Canada

**Keywords:** magnetic stimulation of the brain, CNP signal, Hodgkin and Huxley neuron model, feed-forward neuron network, pulsed magnetic fields

## Abstract

Several experimental results published in the literature showed that weak pulsed magnetic fields affected the response of the central nervous system. However, the specific biological mechanisms that regulate the observed behaviors are still unclear and further scientific investigation is required. In this work we performed simulations on a neuronal network model exposed to a specific pulsed magnetic field signal that seems to be very effective in modulating the brain activity: the Complex Neuroelectromagnetic Pulse (CNP). Results show that CNP can silence the neurons of a feed-forward network for signal intensities that depend on the strength of the bias current, the endogenous noise level and the specific waveforms of the pulses. Therefore, it is conceivable that a neuronal network model responds to the CNP signal with an inhibition of its activity. Further studies on more realistic neuronal networks are needed to clarify if such an inhibitory effect on neuronal tissue may be the basis of the induced analgesia seen in humans and the antinociceptive effects seen in animals when exposed to the CNP.

## Introduction

The nervous system is one of the most studied systems under the action of exogenous electromagnetic fields (Espinosa et al., 2006; Marchionni et al., 2006; Platano et al., 2007; Apollonio et al., 2013; Di Lazzaro et al., 2013). Particularly, experimental and clinical studies that explore the effects of the stimulation of the Central Nervous System (CNS) with weak Magnetic Fields (MF) (i.e., with amplitude up to a few mT) and with a low frequency content (Extremely Low Frequency, ELF, 0–500 Hz) have yielded some evidence of functional changes in excitable biological tissues, as recently discussed in Di Lazzaro et al. (2013). Main results of such a review show: (i) effects of ELF-MFs on the distribution and functionality of cell membrane receptors such as adenosine ones; (ii) the influence on intracellular Ca^2+^ signaling and homeostasis and their correlation with neural stem cell proliferation and differentiation; (iii) changes in electrical activity on the intact human brain and, consequently, changes in neuronal functions as motor control, sensory perception, cognitive activities, sleep, and mood. Pulsed Electromagnetic Fields (PEMFs) have been evidenced as a particularly effective subset of ELF signals; in experimental studies of the last years, many types of PEMFs that differ in frequency content and in waveform, have been used to obtain different physiological effects (Di Lazzaro et al., 2013).

Among these pulsed signals one of the most studied is the Complex Neuroelectromagnetic Pulse (CNP™, Baylis Medical Inc., Canada), specially designed to interact with the neurophysiology of biological systems. It has been shown that exposure of volunteers to the CNP improves their standing balance (Thomas et al., 2001a) and can induce changes in EEG activity (Cook et al., 2004, 2005, 2009). But, more interesting, it has been shown that this signal is able to reduce nociception in land snails (Thomas et al., 1997, 1998) and in mice (Shupak et al., 2004a). In humans (Shupak et al., 2004b, 2006; Thomas et al., 2007) it induces a significant analgesic effect, showing potential as a new modality for the treatment of chronic pain.

Despite the amount of experimental evidence showing the effectiveness of this signal in modulating brain activity (Robertson et al., 2010), how the CNP acts on the neuronal response remains largely unknown.

A first attempt in trying to deepen the understanding of the neuronal functioning under the stimulation of the CNP has been made by Stodilka et al. (2011), who have studied the effects of CNP acting on a cortical network model with 1000 neurons synaptically connected. Each node of the network was represented by the Izhikevich model (Izhikevich, 2003), which is a simplification of the Hodgkin and Huxley (H-H) model (Hodgkin and Huxley, 1952). The paper highlighted an effect of synchronization of the network with signal levels (5 mV) higher than the ones used in the experiments. Although this is a first attempt to model the possible effects of the CNP, it does not specifically provide a framework for understanding how the CNP induces antinociceptive and analgesic effects.

The nociception is a complex process that involves many structures at different levels of the nervous system, both in the spinal cord and in the cerebral areas (Basbaum et al., 2009). The analgesic effect acts in this process and may therefore happen at any of these levels; we chose to focus our attention on the influence that the exposure can have on the activity of cortical structures, because they are the ones that are directly exposed by the magnetic fields.

One way to model neuronal functioning, widely used in literature, is the use of simple H-H representation of neurons. This approach represents a good compromise to design network models of the CNS, since H-H neuron modeling permits to obtain precise single-cell models that capture dynamical intrinsic properties of the neurons, but also allows reasonably fast and efficient simulations (Paffi et al., 2006, 2013; Liberti et al., 2009). Recently Pospischil and colleagues have shown how it is possible to obtain a model of a cortical neuron starting from the equations of simple or augmented H-H (Pospischil et al., 2008).

Aim of this paper is to study, with a modeling approach, the effect of the CNP signal on a simplified model of a neuronal network, in order to investigate both model's responses different from the synchronization effect observed in Stodilka et al. (2011) and sensitivity to values of the transmembrane potential lower than the 5 mV applied (Stodilka et al., 2011).

For this reason, and focusing our interest on the CNP's ability to interfere with neuron activity we have started to study the CNP's interaction with a simple H-H neuronal network model (Camera et al., 2013). The network is a bilayer feed-forward network that overall consists of 26 neurons and that is a simplified topology aimed to represent the feed-forward structure of the cerebral cortex (Adair, 2001), in which each primary neuron is modeled using the H-H representation, with a slightly supra-threshold bias current. This condition could be representative of a pathologic hyperactivity of the neuronal system associated with the sensation of pain. Moreover, since the simple H-H model does not describe the stochastic behavior of neurons, we have introduced in our network model a term that takes into account the endogenous noisy environment of the neurons.

While in Camera et al. (2013) we only showed that CNP, modeled with a transmembrane voltage values ranging from 0.1 to 1 mV, is able to induce neuron silencing, in this paper we propose a deeper and systematic analysis of this kind of effect, aiming to confirm this silencing response of the model under many simulation conditions in terms of biasing current, endogenous noise and signal amplitude. Moreover, we performed simulations using “ad hoc” modified versions of the CNP signal, in terms of both the waveform and duration of each pulse and in terms of the order of the time lags within and between the bursts. This type of analysis, performed systematically for the first time, may help in understanding which features of the signal are the most important for the silencing effect and, in perspective, may become the basis for modifications to the CNP waveform itself.

## Materials and methods

### The CNP signal

**Figure 1:**
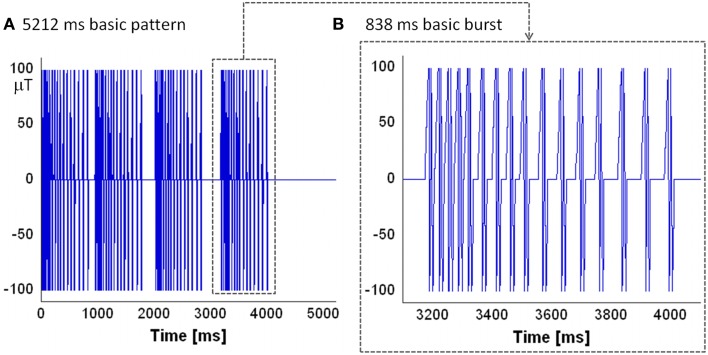
**The evolution in time of the CNP: (A) the basic pattern lasting 5212 ms that is organized in four bursts**. Between bursts there are varying refractory periods of 110, 220, 330 ms followed by a longer fourth one of 1200 ms which begins another basic pattern, repeated for 15 min; **(B)** the basic burst of the pattern: it is composed by a sequence of 16 couples of biphasic waveform whose repetition frequency decreases in time.

CNP is a low power magnetic pulsed signal [typical peak amplitude of about 100 μT (Thomas et al., 1997)]; Figure 1 shows the evolution in time of the CNP signal as taken from the patent [patent number: US6234953 B1 (Thomas et al., 2001b)]. Each pulse of the sequence consists of a couple of biphasic waveforms similar to a neuron's action potentials; the pulses in a burst are similar, but not exactly equal to each other. The signal is organized in bursts with each burst lasting 838 ms (Figure 1B). Both the repetition frequency of the bursts and the repetition frequency of the pulses in a burst are not fixed as they decrease in time. The time interval between a pulse and another is called the latency period, while the time between one burst and another is called the refractory period. The typical basic pattern for the CNP is the one depicted in Figure 1A: four bursts followed by four different refractory periods lasting in order 110, 220, 330, and 1200 ms; so overall the CNP lasts 5212 ms. It is repeated, in experimental conditions, for about 15 min or more (Thomas et al., 1997; Cook et al., 2004).

### The neuronal network model

**Figure 2:**
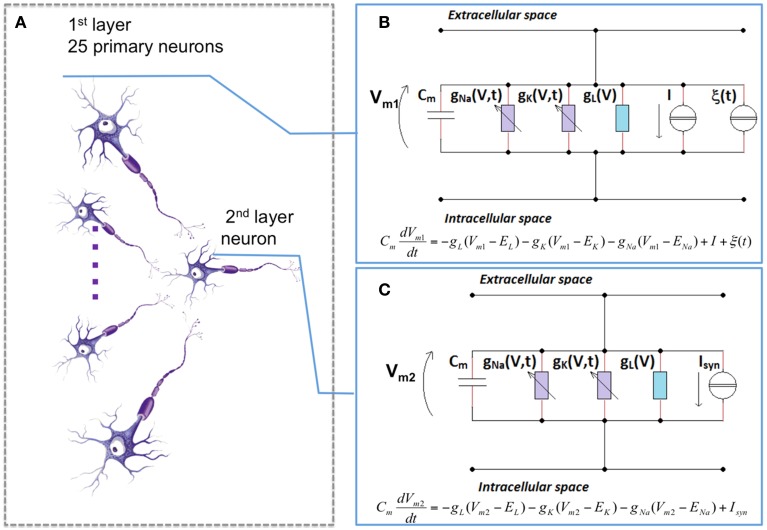
**The neuronal network model. (A)** the topology of the feed-forward network: 25 primary neurons synaptically connected to a coincident secondary one. Each neuron of the first **(B)** and of the second **(C)** layer was described with the H-H formalism. For the first layer **(B)**, no synaptic inputs are considered (*I*_*syn*_ = 0), but there is a current *I*, that accounts for sensory excitation set on three different suprathreshold values (6.5, 6.7, and 7 μA/cm^2^), and a stochastic term (ξ(*t*)) that accounts for the endogenous noise. For the second layer **(C)**, only the synaptic input is considered.

All cortical connections are modeled through a feed-forward structure that consists of two layers, in which 25 primary neurons are linked to a coincident secondary neuron with AMPA (α-Amino-3-hydroxy-5-methyl-4-isoxazolepropionic acid) excitatory synapses (Figure 2A) (Paffi et al., 2013).

Each neuron of this network is considered as an isopotential compartment, and the neuron electrical activity was described with the H-H formalism (Hodgkin and Huxley, 1952), as depicted in Figures 2B,C. In this model, the neuronal membrane is represented by an electrical equivalent, in which the balance of the currents per unit area is given by:

$$\begin{array}{l} C_{m}\frac{dV_{m}}{dt}=\;-g_{l}\left(V_{m}-E_{l}\right)-g_{K}\left(V_{m}-E_{K}\right)\\ \;-\;g_{Na}\left(V_{m}-E_{Na}\right)+I+I_{syn} \end{array}$$

where *C*_*m*_ is a capacitor that takes into account the dielectric properties of the membrane phospholipidic bilayer, *V*_*m*_ is the membrane potential, *g*_*Na*_, *g*_*K*_, *g*_*l*_ are sodium, potassium and leakage conductances per unit area, respectively, and *E*_*Na*_, *E*_*K*_, *E*_*l*_ the reversal potentials of the corresponding currents. Finally, *I*_*syn*_ and *I* are the input currents for the model. These two inputs terms control the transition between the resting state and the firing activity of the neuron. In particular, there exists a threshold value (that, for a H-H model is equal to 6.3 μA/cm^2^) above which the neuron starts its firing activity. The values of *I*_*syn*_ and *I* differ according to the layer to which the neuron belongs (Giannì et al., 2007; Paffi et al., 2007). Neurons in the first layer, in fact, have no synaptic inputs (*I*_*syn*_ = 0) but have *I* ≠ 0 to account for sensory excitation; specifically, three values for this stimulation current have been used (6.5, 6.7, and 7 μA/cm^2^), that means that we stimulate the neuron with slightly supra-threshold currents. Such currents can represent the overall out-of-equilibrium condition (Basbaum et al., 2009) of the sensory/neuronal system afferent to the neuronal network here adopted. As a whole it can be considered a simplified model of the portion of the CNS perceiving the pathological state. Moreover, to account for the intrinsic stochasticity of synaptic input stimuli on the primary layer, a random term ξ(*t*) was added to this current; this term was represented by a Gaussian noise having mean value equal to 0 and variance D, whose chosen values are 0.10, 0.20, 0.25, and 0.30 μA^2^/cm^4^ (Figure 2B).

Conversely, the secondary neuron is excited only by the 25 neurons of the first layer, so for this neuron *I* = 0, while *I*_*syn*_ is given by:

$$I_{syn}=1/25\sum_{i\;=\;1}^{25}I_{syn}^{i}$$

where I^i^_*syn*_ is the contribution of the *i*-th primary neuron that can be calculated considering:

$$I_{syn}^{i}={\bar{g}}_{syn}r(V-E_{AMPA})$$

where g_*syn*_ is set to 0.6 μA/cm^2^, *r* is the gating for the synaptic link, *V* is the membrane potential of the secondary neuron, and E_AMPA_ = 0 mV (Figure 2C).

Numerical simulations have been performed implementing this model in C++ environment using the direct Euler integration method with a time step of 10 μs.

### The introduction of the signal in the network model

Since the CNP is a magnetic signal, we can assume an inductive coupling mechanism inducing an electric field in the brain tissue. In turns, such a field determines a transmembrane potential (Merla et al., 2011, 2012; Denzi et al., 2013), so the term that accounts for the exogenous magnetic field was introduced in the network as an additive component over the membrane potential proportional to the time derivative of the CNP (CNP'), which is similar to the approach taken by Stodilka et al. (2011). For this reason, such an additive term can be represented by a voltage generator, in series with the current branches described above (Giannì et al., 2005, 2006).

We already have seen (Camera et al., 2013) that with a maximum of the absolute value of the input signal (max|CNP′|) that ranges from 0.1 to 1 mV, the CNP is able to lead the neurons in a state of resting, so we performed additional simulations in which the max|CNP'| reaches a maximum of 8 mV.

The signal is applied only on the 25 primary neurons (Giannì et al., 2007; Paffi et al., 2007, 2013). The secondary one is not exposed in order to decouple the effects due to the signal from those due to the synergic action of the neurons of the first layer, focusing, in a first instance, only on the latter.

Since the original sequence taken from the patent (Thomas et al., 2001b) was sampled at 1 ms, we upsampled and interpolated the signal to obtain a time step of 10 μs; this upsampling is necessary because changes in neuronal behavior occur much faster than 1 ms.

Then, we filter the digital sequence in order to remove frequencies that were not intended to be present in the original waveform. The filter used is a fifth-order Butterworth filter with a corner frequency of 500 Hz.

In order to investigate whether the silencing effect observed in Camera et al. (2013) is due to the particular waveform of CNP pulses, or to the sequence of lags between each pulse (the latency periods) and each burst (the refractory periods), we performed simulations modifying the CNP signal, in terms of the waveform of each pulse and in terms of the duration of the latency and refractory periods.

Specifically, to analyze the role of the pulse waveform on the neuron silencing, because of the dissimilarities between the pulses, we used signals where the *i*-th pulse (*i* = 1:n) replaced all the other n-1 pulses in the burst, obtaining an overall signal composed only by the *i*-th pulse waveforms.

We also performed simulations in which we inverted the progression in time of the durations of the latency periods, and the durations of the refractory periods, in order to understand if the silencing effect can be due to the particular order of the progression of these lags.

## Results

### Effect on the primary neurons

#### Silencing effect of the CNP

**Figure 3:**
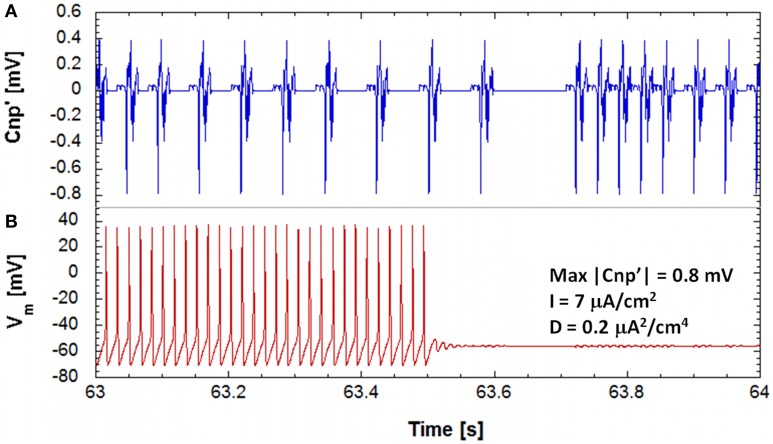
**The evolution of the transmembrane potential of a stimulated neuron (B), on the bottom and the corresponding evolution in time of the input signal (A), on the top during 1 s of exposure, for a simulation on a neuronal network with a polarization current of 7 μA/cm^2^, a noise variance of 0.2 μA^2^/cm^4^ and signal's amplitude of 0.8 mV**. It can be seen that the neuron is brought to a state of resting.

For given sets of the studied parameters (bias current and noise intensity), the main effect of a CNP signal, with a sufficiently high intensity, is a persistent stop of firing observed on one or more of the 25 primary neurons of the network. As example of this silencing effect on the primary neurons of the network, Figure 3 reports the time behaviors, 1 s long, of the applied signal, i.e., the time derivative of the CNP (Figure 3A), and of the correspondent membrane voltage of one neuron (Figure 3B). The neuron silencing begins after a certain time from the application of the signal (see Figure 3) and lasts for the whole simulation duration (15 min), with slight oscillations around the resting potential (−60 mV); even if the signal is removed (data not shown), the neuron does not start to fire again, at least for the durations observed in our simulations, i.e., several minutes. This means that the CNP signal can persistently bring the supra-threshold neuron into a stable resting state, consistent with its analgesic effect experimentally observed (Shupak et al., 2006; Thomas et al., 2007).

**Figure 4:**
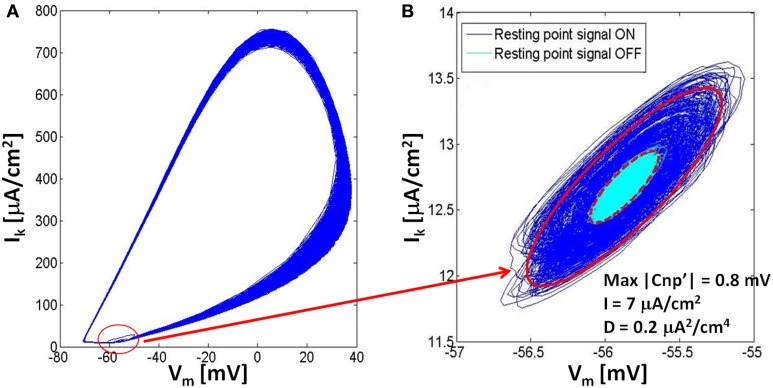
**The rappresentation in the phase plane of the dynamics of a neuron for a stimulation current *I* = 7 μA/cm^2^, noise variance *D* = 0.2 μA^2^/cm^4^ and signal's amplitude of 0.8 mV. (A)** Both the firing state (limit cycle) and the resting state (small elliptic trajectories) are represented in the phase plane; **(B)** Comparison of the resting state in the phase plane with the application of the signal (dark blue) and without it (light blue); we can see that the adding of the CNP doesn't affect the position of the resting state, but increases the axes of ellipses that contain the 99.7% of the trajectories (red lines).

To investigate these oscillations around the resting state, the same trace reported in Figure 3B has been analyzed on a phase plane where the potassium current is represented against the membrane voltage (Figure 4). In Figure 4A the whole trace is reported, including the three time periods where the signal is on and the neuron fires, the signal is on and the neuron is silenced, and the signal is off and the neuron remains silenced. The biggest closed trajectories of Figure 4A represent the first time period when the neuron is still active and lies in the limit cycle; the other two time periods produce the small elliptic trajectories highlighted with a red circle in Figure 4A and reported in detail in Figure 4B. The blue lines are the trajectories, around the resting point, in the presence of signal and noise, whereas the cyan lines represent the oscillations due to the noise alone, when the signal has been removed (Figure 4B). The red lines in Figure 4B represent the contour of the elliptic areas including the 99.7% of the trajectories in the presence (solid line) and in the absence (dashed line) of the signal, respectively.

As evident from Figure 4A, when the signal is applied, the neuron system undergoes a state transition from the limit cycle to the resting state, so that both states coexist in the same phase diagram. Looking at the resting state (Figure 4B) during and after the signal application, it is evident that the signal does not affect either the centers of the ellipses nor their direction and eccentricity, but increases the ellipses axes, with respect to the case in the absence of signal, in a way dependent on the signal amplitude. For example, for the exposure conditions of the Figure 4, the major axis of the ellipse increases 213%, while the minor one increases 207%; when the signal amplitude is decreased to 0.7 mV, also these percentages decrease: the major axis to 179%, while the minor one to 173%. On the other side, with higher noise values (i.e., 0.3 μA^2^/cm^4^) and the same signal amplitude (0.7 mV), we observed further decreases (124% for the major axis, 121% for the minor one), and this is because, increasing noise variance from 0.2 to 0.3 μA^2^/cm^4^, the ellipse of the unexposed trajectory becomes bigger, partially masking the enlarging effect due to the presence of the signal that becomes less noticeable.

This suggests that, for a bias current of 7 μA/cm^2^, the signal application makes the system escape from the limit cycle and oscillate around its own resting state. However, these oscillations are too weak to bring back the system into the attraction basin of the limit cycle. When the signal is removed, even smaller random fluctuations, induced by noise, are present around the baseline, so that the system is likely to remain silenced for an indefinite time, unless an external event occurs, in agreement with the long-lasting analgesic effect experimentally observed. This behavior is also coherent with the bifurcation theory applied to the H-H neuron model (Izhikevich, 2000), indicating that, for a bias current between 6.3 and 9.8 μA/cm^2^ (Hassard, 1978), a stable oscillation and a stable resting state coexist and a sufficiently high perturbation may induce a transition among them.

#### Silencing times

**Figure 5:**
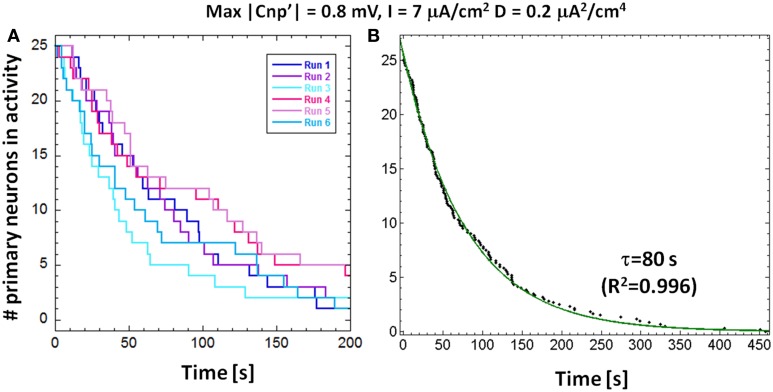
**(A)** Number of primary neurons in activity over time for a stimulation current *I* = 7 μA/cm^2^, noise variance *D* = 0.2 μA^2^/cm^4^ and signal's amplitude of 0.8 mV for six different runs. **(B)** The number of primary neurons in activity averaged over the six runs; by fitting these data with a decreasing exponential, we obtained the time constants of decay.

As observed from Figure 3, the neuron response to the signal is not instantaneous, but a time lag occurs between the signal application and the neuron silencing. For fixed values of the bias current and the signal amplitude, the silencing time is not the same for all the 25 primary neurons, due to the stochastic behavior introduced by the presence of noise. In Figure 5A, we plotted the number of active primary neurons against the time course of the simulations for six different runs performed with the same exposure conditions (*I* = 7 μA/cm^2^, *D* = 0.20 μA^2^/cm^4^ and signal's amplitude of 0.8 mV). We observed, for each run, a decreasing trend, as in Camera et al. (2013) that means that the neurons are not silenced simultaneously. We averaged these trends to obtain the mean number of active primary neurons over the six runs and fitted these data using an exponential decay with a time constant τ (Figure 5B), representing the time interval, after the signal application, when the active primary neurons are reduced to 1/e (approximately 9 active neurons).

This calculated time constant can be used as a measure of the CNP silencing efficiency, especially in those cases when two different CNP signals induce the complete silencing of the first layer of the network during the 15 min of simulation. Moreover, the exponential silencing trend allows us to predict the exposure duration after which the percentage of active primary neurons is reduced below a well-defined threshold, even if it is longer than the simulation time. A parallel interpretation of the exponential decay, if normalized to the total number of primary neurons (25), is as a reliability function, i.e., the probability for a primary neuron to remain active during the time interval between 0 and t. Therefore, the shorter the time constant τ, the lower the probability to find a firing neuron after a well-defined time interval from the signal application.

### A systematic analysis

#### Dependence on signal intensity and noise

The number of silenced primary neurons during the observation time and, in an analog way, the silencing time constant defined in Section Silencing Times, depends on the bias current, the noise intensity and the amplitude of the CNP'. An exhaustive analysis of these dependencies is reported in Tables 1–3, where the percentage of silenced neurons over the 25 of the primary layer averaged over six runs and the silencing time constant τ (calculated as described in Section Silencing Times) are reported for each combination of the noise intensity (D) and CNP' amplitude. For the τ values reported, the goodness of fit is always better than *R*^2^ = 0.95 except for the cases in which the percentage of silencing is less than 5%, where we have few primary neurons silenced and so few data points for the fitting. The CNP' amplitude is defined as the maximum of the absolute value of the CNP'. Such dependencies are shown for each of the considered supra-threshold bias currents: *I* = 6.5 μA/cm^2^ (Table 1), *I* = 6.7 μA/cm^2^ (Table 2), and *I* = 7.0 μA/cm^2^ (Table 3).

**Table 1:**
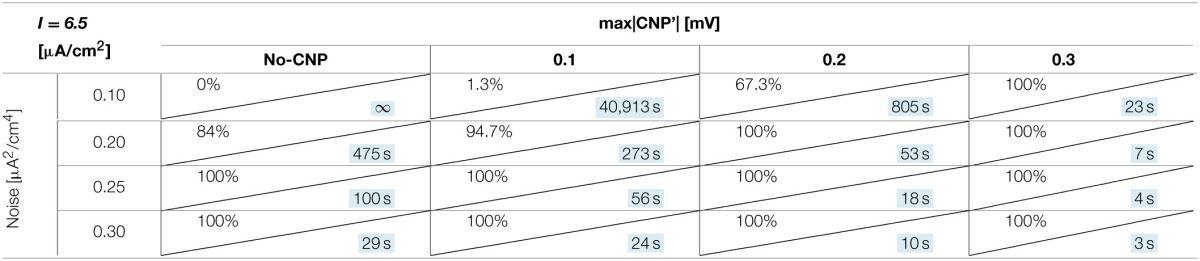
**Percentage of primary neurons silenced and relative silencing constant time (colored in light blue) varying noise variance and signal amplitude for a bias current of 6.5 μA/cm^2^**.

**Table 2:**
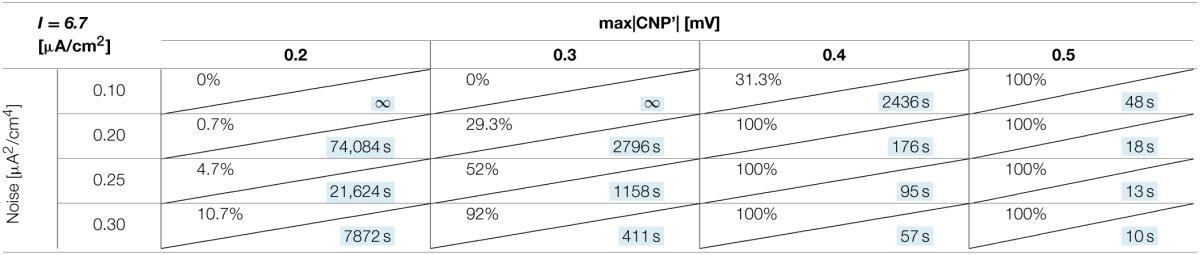
**Percentage of primary neurons silenced and relative silencing constant time (colored in light blue) varying noise variance and signal amplitude for a bias current of 6.7 μA/cm^2^**.

**Table 3:**
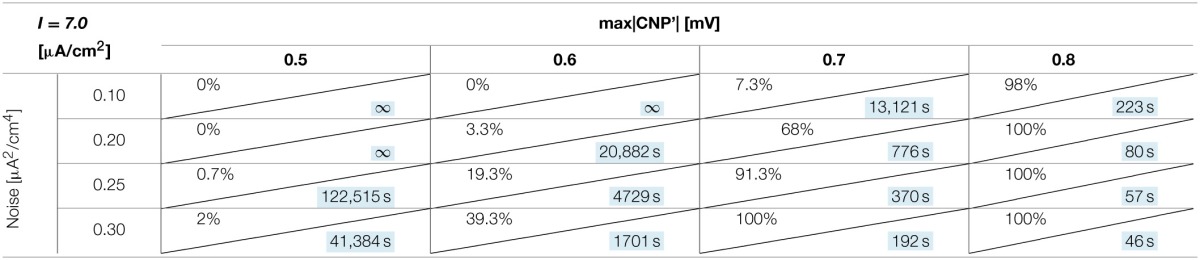
**Percentage of primary neurons silenced and relative silencing constant time (colored in light blue) varying noise variance and signal amplitude for a bias current of 7 μA/cm^2^**.

Before considering the effect of the signal, it should be noticed that, for the bias current closest to the threshold for the appearance of a periodic limit cycle (*I* = 6.5 μA/cm^2^), the noise alone, with intensity equal to or above 0.2 μA^2^/cm^4^, can silence the neurons (second column of Table 1). Indeed, for the noise levels of 0.25 and 0.30 μA^2^/cm^4^, all the primary neurons are silenced in the first 15 min of exposure, with a time constant that decreases with increasing noise intensities. The same effect is not present for the higher levels of bias current, at least for the considered noise intensities.

Looking at the Tables relative to each bias current, there exists a value of the signal above which the silencing effect starts to be observed during 15 min of exposure (percentage of silenced neurons higher than 0) and the closer the current to the threshold, the higher the neuron's sensitivity to the signal. As examples, for *I* = 6.5 μA/cm^2^ (Table 1) and *D* = 0.10 μA^2^/cm^4^, a signal of 0.1 mV is sufficient to induce the neuron silencing, while 0.4 and 0.7 mV are necessary when the bias currents are *I* = 6.7 μA/cm^2^ (Table 2) and *I* = 7.0 μA/cm^2^, respectively. Analogously, the signal level needed to obtain 100% of silencing increases with the bias currents. As already noticed in Section Silencing Times, when 100% of primary neurons is silenced, the time constant is a suitable parameter to compare the silencing efficiency of the signal in different simulation conditions. Noticeably, by increasing the signal intensity, a shorter time is sufficient to obtain silencing.

There is also a cooperative effect of noise in the silencing action: generally, as the noise increases, one can observe an increase in the percentage of silenced neurons and/or a decrease in the silencing time constant.

#### Dependence on the CNP waveforms

In order to investigate what are the peculiar features of the CNP that induce the neuron silencing, we performed simulations using “ad hoc” modified CNP signals, as described in Section The Introduction of the Signal in the Network Model.

In Table 4, the actual CNP and the sequences are compared in terms of percentage of silenced primary neurons and silencing time constant τ for fixed neuron parameters (*I* = 7 μA/cm^2^, *D* = 0.20 μA^2^/cm^4^) and for different signal amplitudes. In Table 4, CNP*i* (*i* = 1, 9, 15) means that we obtained the modified sequence by repeating always the same pulse, the *i*-th, and maintaining the original latency and refractory periods.

**Table 4:**
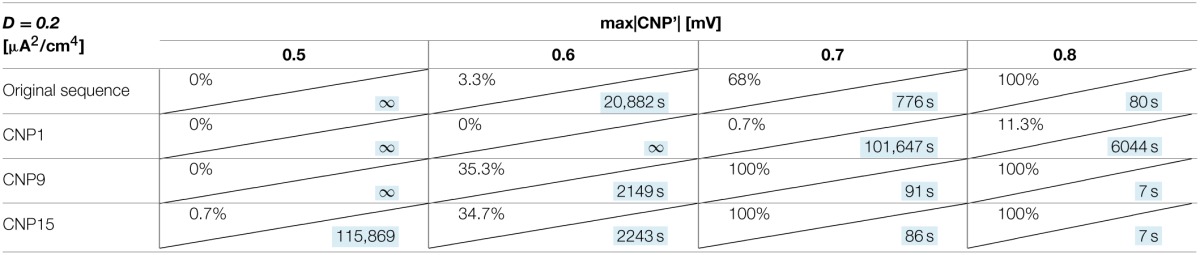
**Percentage of primary neurons silenced and relative silencing time constant (colored in light blue) varying CNP sequence and signal amplitude for a bias current of 7 μA/cm^2^ and a noise variance of 0.20 μA^2^/cm^4^**.

Results of Table 4 show that, for a signal amplitude of 0.5 mV, the considered sequence are not very effective in silence the neurons but, as the amplitude increase, the differences among signals become evident. In particular, CNP9 and CNP15 are more efficient than the actual CNP in silencing the primary neurons, while CNP1 starts to silence only if its amplitude is equal to 0.7 mV.

Thus, the silencing effect seems to be waveform dependent, since some pulse waveforms have shown to be more effective than others. On the contrary, the neuron response does not significantly change (variations of τ always below 10%) when the durations of the latency or of the refractory periods are inverted inside the signal sequence (data not shown).

### Effect on the secondary neuron

When the CNP is applied to a primary neuron of the network, the observed effect is a sharp and irreversible transition from firing to resting, after a random time lag from the signal application. Considering all the 25 neurons of the primary layer, one can observe a silencing trend that follows an exponential decay, as described in Section Silencing Times. This decrease in the number of active primary neurons over time implies the onset of a very irregular activity in the secondary neuron that eventually ceases to fire in turn. These activities can be described as a sequence of periods of reversible silencing, which will be referred to as “partial silencing.”

**Figure 6:**
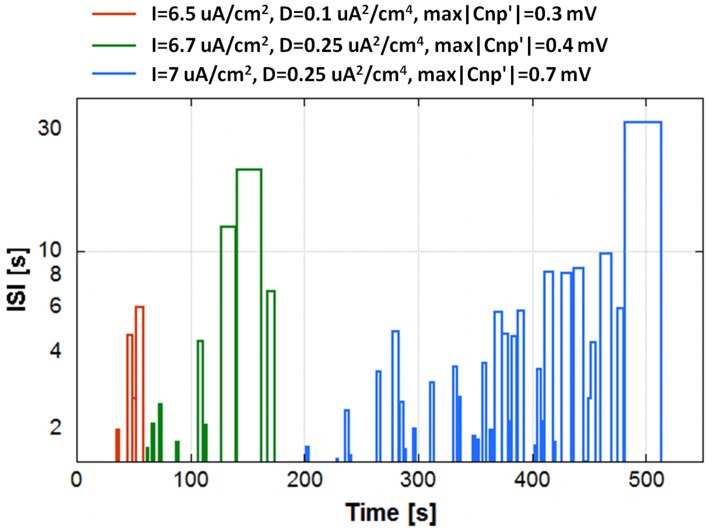
**The “partial silencing” of the secondary neuron in terms of Inter Spike Interval (ISI) as a function of time for a run of three different conditions of exposure: *I* = 6.5 μA/cm^2^, *D* = 0.10 μA^2^/cm^4^, max|CNP'| = 0.3 mV (red line); *I* = 6.7 μA/cm^2^, *D* = 0.25 μA^2^/cm^4^, max|CNP'| = 0.4 mV (green line); and *I* = 7.0 μA/cm^2^, *D* = 0.25 μA^2^/cm^4^, max|CNP'| = 0.7 mV (cyan line)**.

The “partial silencing” of the secondary neuron is shown in Figure 6 using three significant examples. The graph plots the durations of these “partial silencing” in terms of Inter Spike Interval (ISI) against the time instants when they start, for a specific run. Durations below 1.5 s are not plotted, since they can be considered inside the normal variability range of the (ISI), not an actual silencing. The conditions considered in Figure 6 are: *I* = 6.5 μA/cm^2^, *D* = 0.10 μA^2^/cm^4^, max|CNP'| = 0.3 mV (red line); *I* = 6.7 μA/cm^2^, *D* = 0.25 μA^2^/cm^4^, max|CNP'| = 0.4 mV (green line); and *I* = 7.0 μA/cm^2^, *D* = 0.25 μA^2^/cm^4^, max|CNP'| = 0.7 mV (cyan line). In the first case, a significant “partial silencing” begins at 30 s, when the primary neurons still in activity are 8, and this silencing becomes definitive at 60 s, when only three primary neurons are active, in the second case, “partial silencing” events start after 50 s, when 11 primary neurons are active; as long as the primary neurons become silent following the time constant τ, the silencing period tends to become longer and the irreversible silencing occurs with two primary neurons in activity, i.e., at around 180 s. In the last case, the time lag between the beginning of the “partial silencing” (200 s corresponding to 13 active primary neurons) and the irreversible silencing (512 s) is much longer than in the previous cases, in agreement with the higher time constant (260 s). Interestingly, in all cases, the phenomenon of “partial silencing” requires that slightly more than half of the total primary neurons are silenced; therefore, in a more complex system such as the network, the cooperative action of multiple neurons determines a considerable effect on the secondary neuron without necessarily having a consistent effect on the primary neurons.

### High intensity CNP: synchronization

In this section we study the effect of the CNP on one primary neuron when the intensity is increased up to 8 mV. Results indicate that a complete different effect arises: increasing the signal amplitude, the neuron's activity starts to synchronize with the signal, in agreement with the theoretical results reported in Stodilka et al. (2011).

**Figure 7:**
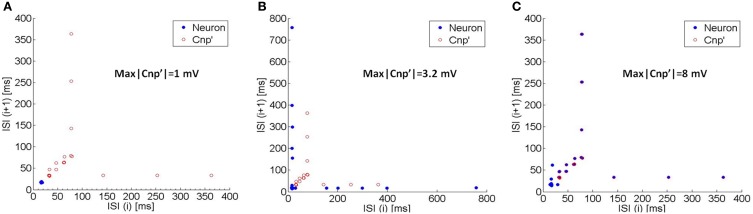
**The Poincaré maps (i.e., the i-th ISI vs. the previous one) of the neuron's activity (blue circles) and of the CNP' (red circles) for three different exposure conditions: (A) max|CNP'| = 1 mV, (B) max|CNP'| = 3.2 mV, and (C) max|CNP'| = 8 mV**. As the signal's amplitude increases, the neuron's map begins to overlap with the CNP”s one, so the neuron's spikes occur at the same instants of the CNP' pulses (synchronization).

The onset of this synchronization mechanism is evident from the Poincaré maps of Figure 7. The Poincaré maps represent the *i*-th Interspike Interval (ISI) vs. the previous ISI (ISI(*i*+1); ISI(*i*)) (Rasband, 1990); therefore, if the neuron exhibits a regular firing, each ISI is similar to the previous one, so that the neuron activity is represented on the map by a single point lying on the bisector. Conversely, if the neuron spikes are arranged in bursts, they are represented by points lying on a horizontal segment and a vertical one and are symmetric with respect to the bisector.

Due to the typical shape of the CNP', consisting in a quite regular sequence of pulses, it can be efficiently represented in a Poincaré map if, instead of the ISIs, we consider the time interval between the minimum peaks of the signal. The CNP' on the maps is plotted with the red circles that evidence the typical structure (see Section The CNP Signal) with increasing time lags between the pulses (latency periods) and even longer time intervals between the bursts (refractory periods).

When the CNP' amplitude is 1 mV, the neuron firstly exhibits a quite regular firing (blue circle in Figure 7A), then ceases to fire, in agreement with the silencing effect examined in Section Silencing Effect of the CNP. However, when the signal intensity increases up to 3.2 mV, the blue circles in Figure 7B show a typical bursting behavior, indicating that the neuron alternates periods of silencing with periods of firing activity. With an even higher signal intensity (8 mV), the neuron activity is completely synchronized with the signal, i.e., the spikes occur at the same time instants of the CNP' pulses, as evident from Figure 7C where the blue circles overly the red ones.

Results of this work are particularly interesting because they evidence two different mechanisms of action of the same signal that can be used to induce different effects depending on the chosen intensity.

## Discussion and conclusions

The main effect induced by the CNP signal on the primary neurons of a simple model of feed-forward network is an irreversible silencing (Figure 3) that persists even after the signal has been removed. However, the silencing time is not the same for all the primary neurons of the network but occurs at random time instants after the application of the CNP. If one considers the number of the active primary neurons as a function of the silencing instants, the resulting plot can be well-fitted with an exponential decay (Figure 5B). This function can have a two-fold interpretation: it describes the rate of neuron silencing but also the reliability function, i.e., the probability of finding a neuron active after a well-defined time lag from the signal application. In any case, the time constant τ of the exponential decay can be considered as a quantitative parameter to describe the silencing efficiency of different stimulation conditions. It has been shown that τ depends on the signal amplitude, as well as, on the models parameters: the bias current and the internal noise. In particular, the time constant decreases with increasing signal amplitudes and noise levels and with bias currents that approach the threshold current for the onset of periodic oscillations in an H-H neuron model (6.3 μA/cm^2^). The dependence on noise indicates a cooperative action of signal and noise in the neuron silencing; thus, the physiological presence of noise can significantly reduce the signal level necessary to obtain the desired effect.

An analysis on the phase plane (Figure 4) has shown that, when the silencing occurs, the dynamic system representing the neuron undergoes a state transition, from a stable limit cycle to a stable resting state. Such a transition is more likely to occur as long as the bias current is close to the threshold current (*I* = 6.3 μA/cm^2^). This behavior can be explained with the bifurcation theory (Hassard, 1978; Izhikevich, 2000) indicating that, in a H-H system, if the bias current varies between 6.3 (fold limit cycle bifurcation) and 9.8 μA/cm^2^ (subcritical Adronov-Hopf bifurcation), two stable states coexist: the limit cycle and the resting state (Izhikevich, 2000). This means that, for bias currents in the aforementioned range, if the signal brings the system in the resting state, it stably remains there, unless an intense exogenous stimulation makes the system escape from the attraction basin of the resting state, crossing the instable oscillation orbit on the phase plane. These theoretical explanations seem to have also experimental confirmations (Toups et al., 2012; Meng-Jiao et al., 2014).

Interestingly, the silencing effect seems to be waveform dependent, since some CNP pulses have shown to be more effective than others (Table 4), when all the other conditions are fixed (bias current, signal amplitude, noise intensity). This result indicates a specific interaction between the signal and the modeled neuronal network.

Moving to the secondary layer of the network, the observed effect is a modulation of the firing activity of the neuron, with long and reversible silencing periods (partial silencing) (Figure 6), occurring only when at least one half of the primary neurons is silenced. Therefore, even though the secondary neuron is not exposed, it is strongly affected by the signal and, after an irregular behavior, ceases to fire accordingly to the primary silencing.

These results reveal an overall interaction of the CNP signal with a neuronal network model, with a general inhibitory action when the model is set in a slightly suprathreshold condition. This model response seems congruent with experimental data wherein the application of the CNP induces analgesia in humans and antinociception in snails and mice.

When increasing the CNP amplitude, the observed effect is completely different: the neuron firing begins to synchronize with the CNP pulses. This result agrees with the oscillator theory (Rinzel and Ermentrout, 1998), since the applied signal is strong enough to induce a phase locking in the system oscillation and with results obtained in Stodilka et al. (2011) with a different neuronal network.

We conclude that these results, using a relatively simplified feed-forward H-H network model, justify further studies of the effect of CNP exposures. Specifically more complex and realistic models should be used that represent areas of the brain shown to be affected by CNS exposure and that are involved in the pain perception, such as the insula, the anterior cingulate, the hippocampus and the caudate (Robertson et al., 2010). Moreover, since for some exposure conditions (Shupak et al., 2004b) the signal can interfere also with structures at the level of spinal cord, future works will have to consider also the possibility that the analgesic effect could be induced by the signal directly in the specific inhibitory circuits that are responsible for the “gate control” (Melzack and Wall, 1967) without involving directly the final station of the cortex area.

### Conflict of interest statement

The authors declare that the research was conducted in the absence of any commercial or financial relationships that could be construed as a potential conflict of interest.
